# Succession of Weed Community on Wheat Lands in the Past 25 Years: A Case Study in Eastern China

**DOI:** 10.3390/biology14080943

**Published:** 2025-07-27

**Authors:** Guoqi Chen, Zeyue Huang, Jiahao Xue, Feng Zhu, Yang Chen, Yunfei Wu

**Affiliations:** 1 Jiangsu Key Laboratory of Crop Genetics and Physiology/Jiangsu Key Laboratory of Crop Cultivation and Physiology, Research Institute of Rice Industrial Engineering Technology, Agricultural College of Yangzhou University, Yangzhou 225009, China; hh1003741822@163.com (Z.H.); 18205251761@163.com (J.X.); yzuchenyang@163.com (Y.C.); 2 Jiangsu Co-Innovation Center for Modern Production Technology of Grain Crops, Yangzhou University, Yangzhou 225009, China; 3 Jiangsu Green Food Office, Nanjing 210036, China; jszbczf@163.com; 4 College of Bioscience and Biotechnology, Yangzhou University, Yangzhou 225009, China; 006949@yzu.edu.cn

**Keywords:** biological homogenization, broadleaf weed, dominance, field survey, grassy weed, historical data, species richness

## Abstract

Weeds represent increasingly troublesome threats to modern wheat production. Jiangsu Province, China, is one of the largest wheat-planting areas, characterized by a high level of agricultural mechanization, in the country. In 2024, we surveyed weed communities of 924 wheat lands from 308 sites in Jiangsu and compared them with historical data surveyed in 1999–2000 in this province. A total of 156 weed species belonging to 103 genera and 39 families were recorded. Compositae and Poaceae showed the highest species richness, with 26 and 23 species, respectively. Poaceae weeds accounted for 54.3% of the overall weeds in dominance values. *Beckmannia syzigachne* showed the highest proportion of the overall weeds in dominance values (19.2%). Compared with the historical data, weed diversity increased by 33.3% in species, and by 62.5% in families; grassy weeds such as *Beckmannia syzigachne*, *Alopecurus japonicus*, and *Alopecurus myosuroides* became more dominant, while several low-growing grassy weeds became less dominant. The dominance of broadleaf weeds, such as *Galium* spp., *Vicia* spp., and *Veronica* spp., decreased greatly, and a clear trend of weed homogenization among different areas and types of wheat lands was observed. This is the first investigation of weed community succession against the background of agricultural modernization in China.

## Simple Summary

Weeds are among the most significant challenges to wheat production. Jiangsu Province, China, is a major wheat-producing region that has undergone a transition from traditional to modern farming methods in recent decades. In 2024, we conducted a field survey on weed communities of 924 wheat fields across the province and identified 156 different weed species. Compared with weed communities of wheat lands in this province surveyed 25 years ago, we observed a substantial increase in weed diversity across species, genera, and families. Some fast-growing grassy weed species have become more widespread and dominant, while several smaller, low-growing ones have declined. Meanwhile, the dominance values of several broadleaf weeds has decreased sharply. Moreover, clear patterns of homogenization in weed communities across different regions and different types of wheat fields were observed. This study represents a typical, systematic investigation of weed communities on wheat lands on a regional scale based on field surveys, against a background of wheat-cultivating systems shifting from traditional to modern methods at a large scale. Our findings provide important information for farmers and policymakers to design more effective weed management strategies for modern wheat-cultivating systems.

## 1. Introduction

Wheat is one of the most important crops in the world, with a total production of 1,163,497,300 tons from an area of 203,470,000 hectares in 2022 [1]. Weeds represent one of the biggest biological challenges to wheat production worldwide, estimated to cause an actual yield loss of 7.7% worldwide [2]. In wheat cultivation, weed management largely relies on applying chemical herbicides. Considering the real expense and time-consuming nature of developing a new herbicide, wheat herbicide active ingredients are limited worldwide [3]. Meanwhile, chemical control strategies frequently fail for multiple reasons. First, weed communities in wheat fields consist of diverse species, which may have different sensitivities to different herbicides; and some weed species may survive and cause significant yield losses [4]. Second, the sensitivity of a particular weed species to certain wheat herbicides usually decreases with increasing growing stages, which may also result in failed chemical control [5]. Third, herbicide efficacy varies greatly with variations in environmental conditions such as temperature, soil moisture, wind and rainy weather, application techniques such as spraying volume, sprayer, and preparation procedures in tank mixtures, as well as field management practices [6]. Fourth, weeds frequently evolve herbicide resistance, which has become one of the biggest challenges in wheat weed management [7]. Moreover, overdosing of chemical herbicides poses significant ecological and food safety risks [8]. Therefore, integrated weed management (IWM) strategies, which combine multiple technologies, have been increasingly emphasized in many wheat-growing regions around the world [9]. A comprehensive understanding of the composition and successional patterns of weed communities in wheat fields is fundamental for developing effective IWM approaches.

Weed communities in wheat fields vary substantially across different climatic zones and agricultural practices. For example, Rassam et al. (2011) reported the impact of diverse crop management practices on weed community composition in wheat fields [10]. Zhang et al. investigated the effects of tillage methods on the weed diversity of winter wheat fields [11], and Seipel et al. examined how cropping systems influence weed communities in winter wheat under varying climatic conditions [12]. However, there remains a lack of large-scale, systematic field survey data, limiting our understanding of weed community structure and succession at regional scales.

Jiangsu Province, China, is one of the world’s major modern wheat-producing regions and faces serious challenges due to weed infestations. Most arable lands in Jiangsu Province follow a wheat–rice or wheat–corn rotation system, with two crop-growing seasons per year. In 1999, Wang et al. surveyed weed communities in wheat lands in Jiangsu by surveying 150 sites with 1500 fields distributed across different areas of the province [13], and reported the occurrence and distribution of 118 weed species, which could be a historical data source for comparing weed communities in different periods. Over the past 25 years, wheat-planting areas increased from 2,251,700 hectares in 1999 to 2,389,520 hectares in 2023, with the average yield increasing from 4755 kg/ha to 5748 kg/ha, suggesting a 21% yield increase [14]. During the past 25 years, wheat cultivation in Jiangsu has transformed from traditional home-run lands to large mechanical farms [15]. In 1999, the total power of agricultural machinery in Jiangsu Province was 27,679,000 kW, which subsequently increased to 53,602,000 kW by 2023, representing a growth of 93.7%. The number of combine harvesters rose from 295 units in 1999 to 173,273 units in 2023, marking an increase of 586.4% [14]. Weed management further tended to rely on chemical control, which reformed the structure of local wheat communities. Thus, wheat lands in Jiangsu could be a typical scenario for understanding weed community succession in the process of mechanization and farming intensification of agriculture in the world. In 2024, we surveyed weed communities of 308 sites with 924 wheat lands across 82 counties in Jiangsu Province. We hypothesize that weed communities were reformed in this province during the development of agricultural mechanization.

## 2. Materials and Methods

### 2.1. Study Area and Period

**Figure 1:**
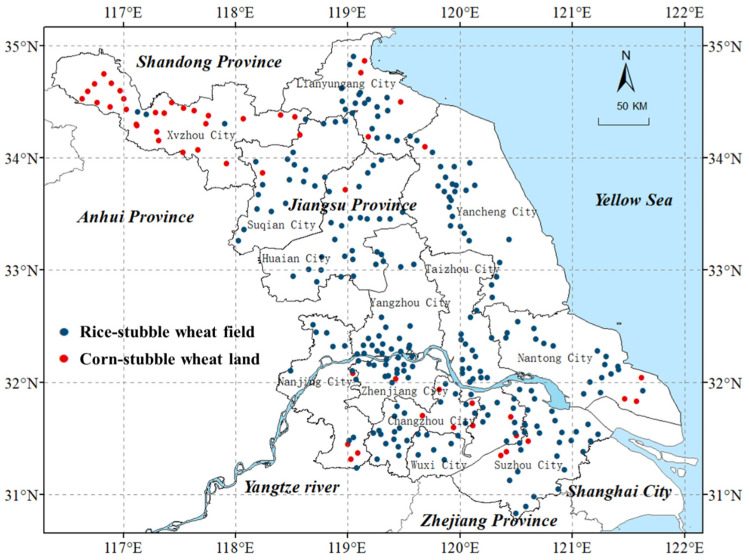
Distribution of the sampling sites.

Jiangsu Province (30°45′ to 35°08′ N, 116°21′ to 121°56′ E) is located in eastern China, covering a total area of approximately 107,200 km^2^ (Figure 1). The province spans from subtropical to temperate climatic zones. According to the Jiangsu Statistical Yearbook 2024 (tj.jiangsu.gov.cn/2024/index.htm) (accessed on 21 March 2025), mean annual temperatures range from 15.4 °C to 18.1 °C; mean temperatures of the coldest month (January) range from 1.6 °C to 6.1 °C; mean temperatures of the hottest month (July) range from 27.8 °C to 29.7 °C; and annual precipitation varies from 898.1 mm to 1631.4 mm, across the 13 cities of this province [14]. In the surveyed wheat fields, rice stubble lands were mostly classified as paddy soils, while corn stubble lands were primarily brown and red soils. The soil pH across the province ranged from 5.61 to 8.31, with an average of 7.02. Overall, soil fertility was relatively high, with sufficient supplies of nitrogen, phosphorus, and potassium [16]. Jiangsu Province is one of the most important winter wheat-producing regions in China, where wheat is usually harvested from late May to early June.

### 2.2. Survey Methods

Field surveys were conducted in May 2024 (Figure 1). All wheat-planting counties in Jiangsu were surveyed, with three to five locations randomly selected for each county. A total of 308 sites were established across 82 counties in Jiangsu Province, and an interval of >10 km was set between adjacent sites. In each site, three wheat lands with the same rotation system (rice stubble or corn stubble) were surveyed. For each wheat land sampled, an area of about 667 m^2^ without field ridges was randomly selected. We walked around the sampling site, and then walked along one diagonal line of the sampled site, and recorded each weed species observed during field walking. At the end of the site field surveying, the average shoot height (>80cm, 20–80cm or <20cm) and coverage of each weed species were visually estimated, which resulted in a dominance value for each weed species in the site (Table 1) [17,18]. The previous crop for each sampled site was determined by checking stubbles and straws on the field. Some weed plants may die before field surveys, while their withered shoots and leaves usually persisted on fields, such as *Capsella bursa-pastoris* (L.) Medik, which were also included in the survey. A total of 251 rice stubble sites and 57 corn stubble sites were surveyed. The family and genus classifications, as well as the morphological identification of each weed species, were determined according to Flora of China [19] (www.iplant.cn/foc) (accessed on 21 March 2025).

### 2.3. Historical Dataset Sources

Wang and Qiang [13] conducted field surveys on wheat weed communities with the above-mentioned visual scoring method in the same province in 1999 and 2000; thus, the dataset reported in their study was cited as the historical dataset. The historical survey covered 150 sites with 1500 wheat fields, recording 118 weed species belonging to 24 families and 78 genera. Historical results were cited from Wang and Qiang [13], which did not show detailed data of weed dominance values of each site. Thus, it was unavailable for us to compare statistically significant differences between results from the historical data and those from our surveys. Moreover, our study surveyed 308 sites with 924 wheat lands; considering the large sampling sizes in both surveys, the results of the mean values could be representative even without SEs.

### 2.4. Data Statistical Analysis

To better compare our results with those of the historical dataset, the average weed dominance value of each weed species at each site was determined based on the dominance values of the three sampled lands at the same site. For each site, longitude, latitude and the type of wheat land (rice stubble or corn stubble) were recorded. The longitude and latitude of each site were determined based on the central point of the site. Weed species with a frequency among 308 sites >10% were designated as common weed species, which included 50 weed species (Table S1). Thus, we set three data matrixes as “site-common weed species dominance value”, “site–dominance value of genus referring to common weed species”, and “site-environmental factors (latitude, longitude and land type)”. Redundancy analysis (RDA) and principal component analysis (PCA) were performed using OriginPro 2024 software. The analysis was based on the default Euclidean distance matrix. Significance of the RDA model was evaluated using permutation tests conducted via the vegan package in R 4.3.0 Independent sample *t*-tests were used for comparing weed dominance values between rice stubble and corn stubble wheat lands conducted in SPSS 16.0. Data were checked for normality and constant variance before analysis, and independent sample *t*-tests in SPSS 16.0 directly indicated significances for both situations of equal variances assumed and equal variances not assumed. Species diversity was determined using the Shannon–Wiener α-diversity index and Pielou’s evenness index [20]. Linear regressions were applied to examine the potential relationships of weed community diversity indices with latitudes and longitudes of sites sampled.

## 3. Results

### 3.1. Composition and Succession in Plant Families

**Figure 2:**
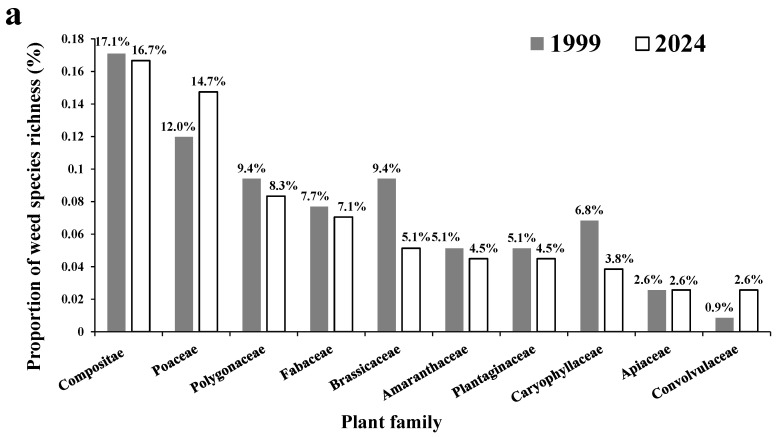

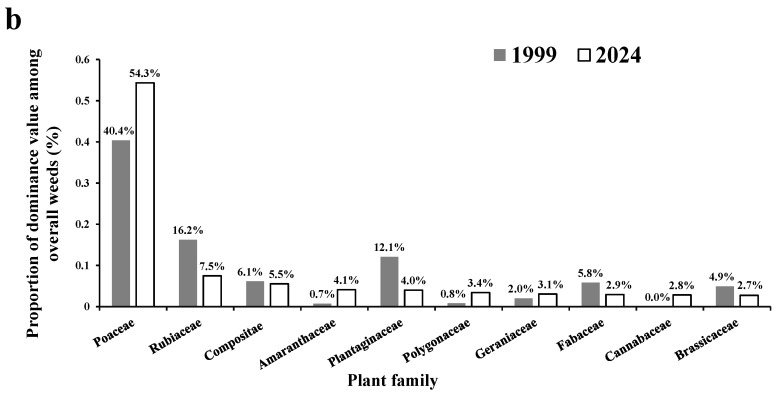
Proportions in species richness (**a**) and dominance value (**b**) of plant families out of overall weeds surveyed. The data were calculated based on the overall data surveyed and thus did not have confidence intervals or standard deviations. For example, each column in the subfigure a showed a percentage of the total number of species of a plant family out of the total number of species in the current or historical data source, which did not have replications as a whole. The same can be seen below.

A total of 156 weed species (Table S1) were recorded from the 308 surveyed sites, belonging to 39 families. An average of 10.9 families per site were recorded. Compositae showed the highest species richness, with 26 species (16.7% out of the 156 weed species), followed by Poaceae and Polygonaceae, with 23 (14.7%) and 13 species (8.3%), respectively. Fabaceae (11 species, 7.1%), Brassicaceae (8 species, 5.1%), Amaranthaceae (7 species, 4.5%), Plantaginaceae (7 species, 4.5%), and Caryophyllaceae (6 species, 3.8%) also contained at least six species (Table S2). Compared with the historical data (Figure 2a), the number of weed families recorded in this study increased by 15. Brassicaceae and Caryophyllaceae showed the most notable decreases in their proportions of weed species richness, by 4.3% and 3.0%, respectively. Poaceae exhibited the greatest increase, by 2.8%.

Regarding the dominance values of weed families (Figure 2b, and Table S2), Poaceae accounted for the highest proportion, with 54.3% of the overall weeds, followed by Rubiaceae (7.5%), Compositae (5.5%), Amaranthaceae (4.1%), Plantaginaceae (4.0%), Polygonaceae (3.4%), and Geraniaceae (3.1%). Compared with the historical data (Figure 2b), Poaceae showed the highest increase in dominance value proportion among all the weed families, by 13.9%. In contrast, the proportions of Rubiaceae and Plantaginaceae declined by 8.7% and 8.1%, respectively.

### 3.2. Composition and Succession in Plant Genera

**Figure 3:**
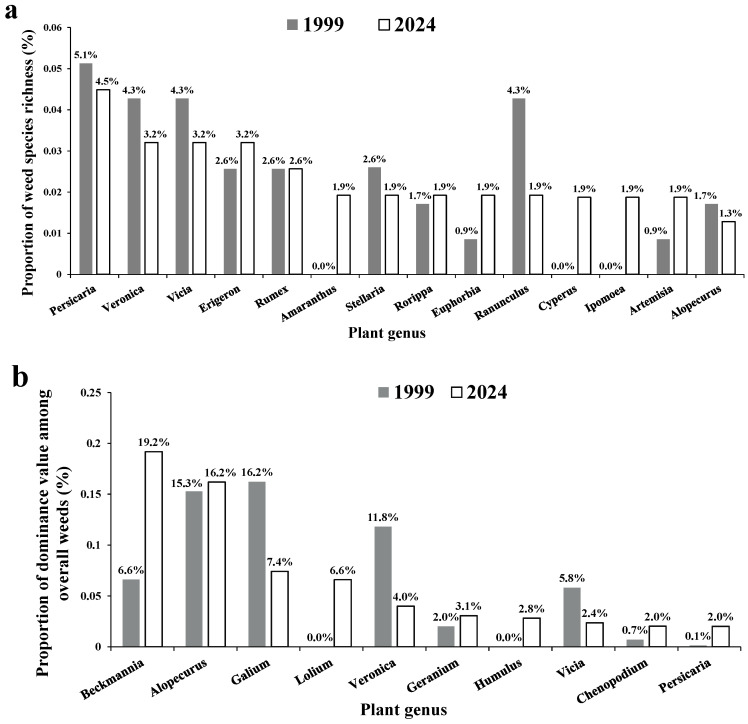
Proportions in species richness (**a**) and dominance value (**b**) of plant genera out of overall weeds surveyed.

A total of 103 genera were recorded in this survey (Table S3), with an average of 17.2 genera recorded per site. *Persicaria* had the highest weed species richness (seven species). *Veronica, Erigeron,* and *Vicia* each recorded five species. *Rumex* recorded four species (Table S3). Compared with the historical data (Figure 3a), the number of weed genera recorded in this study increased by 25. *Amaranthus, Cyperus*, and *Ipomoea* showed the highest increase in the proportion of weed species richness, by 1.9%. *Ranunculus* exhibited the greatest decrease, by 2.4%. *Veronica* and *Vicia* decreased by 1.1%.

Regarding the dominance values of weed genera (Figure 3b and Table S3), *Beckmannia* had the highest proportion out of the overall weeds, accounting for 19.2%, followed by *Alopecurus* (16.2%), *Galium* (7.4%), *Lolium* (6.6%), *Veronica* (4.0%), *Geranium* (3.1%), and *Humulus* (2.8%). Compared with the historical data (Figure 3b), *Beckmannia* exhibited the highest increase, by 12.6%. In contrast, *Galium* showed the greatest decrease, by 8.8%, followed by *Veronica* (7.8%).

### 3.3. Composition and Succession in Plant Species

An average of 18.3 weed species per site was recorded. Compared with the historical data (Table 2), the number of species recorded in this study increased by 39. *Geranium carolinianum* L. showed the highest frequency among the 308 surveyed sites (86.4%), followed by *Galium spurium* L. (76.0%), *Beckmannia syzigachne* (Steud.) Fernald (74.7%), *Veronica persica* Poir. (64.6%), *Humulus scandens* (Lour.) Merr. (53.6%), *Elymus kamoji* (Ohwi) S. L. Chen (52.9%), and *Capsella bursa-pastoris* (L.) Medik. (52.3%). Compared with the historical data, *Geranium carolinianum* showed the highest increase in frequency, followed by *Humulus scandens* and *Elymus kamoji*. In contrast, *Pseudosclerochloa kengiana* (Ohwi) Tzvelev showed the highest decrease in frequency, followed by *Galium tricornutum*, *Erigeron bonariensis*, and *Lithospermum arvense*.

As for dominance values, *Beckmannia syzigachne* showed the highest proportion out of the overall weeds, accounting for 19.2%, followed by *Alopecurus japonicus* Steud. (9.4%), *Galium spurium* (7.4%), and *Lolium multiflorum* Lamk. (6.5%). Compared with the proportions in dominance values of the historical data, *Beckmannia syzigachne* showed the highest increase, followed by *Lolium multiflorum*, *Humulus scandens*, and *Alternanthera philoxeroides*. In contrast, *Veronica persica* showed the highest decrease, followed by *Galium spurium*.

### 3.4. Composition and Succession in Weed Species Groups

**Figure 4:**
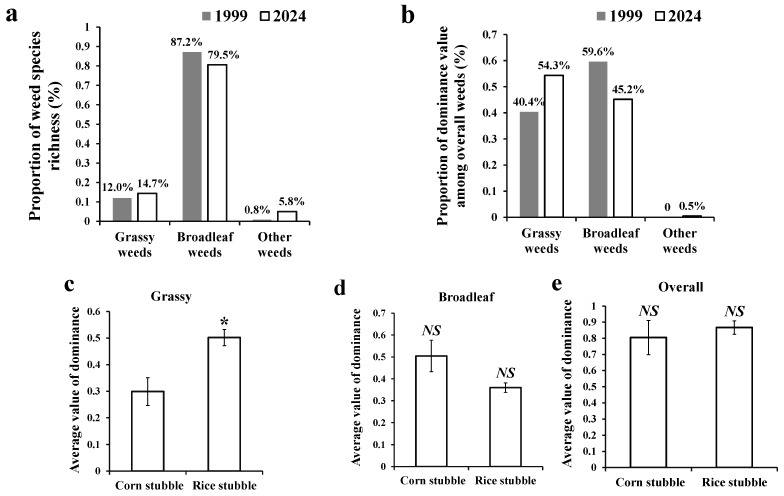
Proportions in species richness (**a**) and dominance value (**b**) of different weed groups out of overall weeds surveyed, and average dominance values of grassy weeds (**c**), broadleaf weeds (**d**), and overall weeds (**e**) of two different types of wheat lands. Note: in (**c**–**e**), values are presented as mean ± SE. *: significant difference, and *NS*: no significant difference between corn stubble and rice stubble fields (independent *t*-test) at *p* < 0.05.

**Figure 5:**
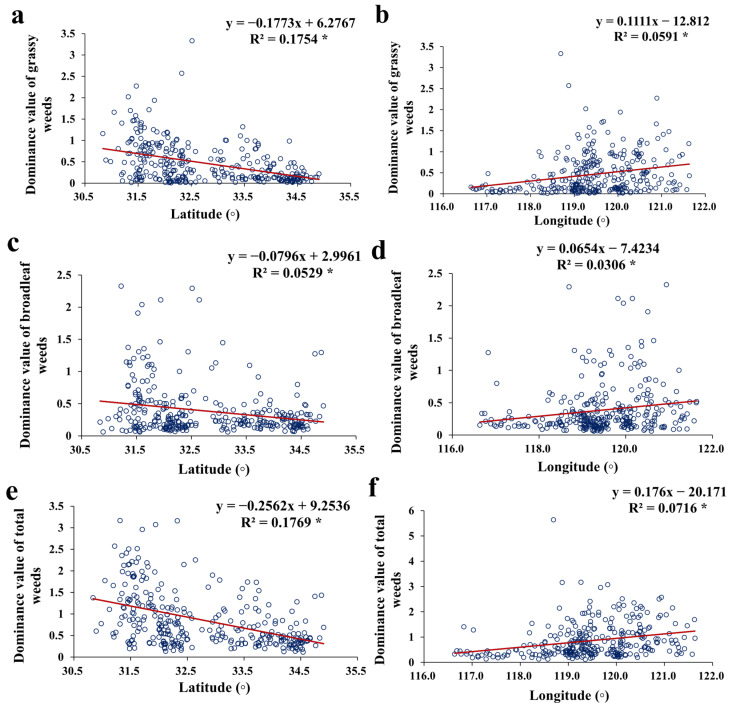
Correlations between dominance values of grassy weeds (**a**,**b**), broadleaf weeds (**c**,**d**), or overall weeds (**e**,**f**) and latitudes or longitudes of wheat land sites surveyed. Note: *: significant correlation at *p* < 0.05.

Among the 156 weed species recorded in this survey, 23 species were grassy (weed species belonging to the family Poaceae, also known as gramineous weeds), accounting for 14.7% of the total weed species; 124 (79.5%) were broadleaf weeds (Figure 4a); and the remaining species were from other weed groups, such as sedge and fern weeds. In terms of the proportion of dominance values out of the overall weeds, grassy weeds made up 54.3% of the total, and broadleaf weeds accounted for 45.2% (Figure 4b). Compared with the historical data, the proportion of grassy weeds increased by 13.9% out of the overall weeds, and broadleaf weeds showed a 14.4% decrease (Figure 4a,b). The dominance value of overall grassy weeds on rice stubble wheat lands was significantly higher (*p* < 0.05) than that on corn stubble wheat lands (Figure 4c), while there was no significant difference (*p* > 0.05) in the dominance value of overall broadleaf weeds (Figure 4d) or overall weeds (Figure 4e). Moreover, the dominance values of grassy weeds, broadleaf weeds, and overall weeds per wheat land were significantly and negatively correlated with latitudes (Figure 5a,c,e), and significantly and positively correlated with longitudes (Figure 5b,d,f).

### 3.5. Influence of Environmental Factors on Weed Communities

An independent sample *t*-test suggested that the average dominance values of 12 weed species were significantly (*p* < 0.05) different between rice or corn stubble wheat lands (Table 3). Specifically, the average dominance values of *Veronica persica*, *Polypogon fugax*, *Descurainia sophia*, *Capsella bursa-pastoris*, *Humulus scandens*, and *Stellaria aquatica* were significantly higher on corn stubble wheat lands, while those of *Beckmannia syzigachne*, *Alopecurus japonicus*, *Echinochloa crus-galli*, *Galium spurium*, *Acalypha australis*, and *Mazus pumilus* were significantly higher on rice stubble wheat lands.

**Figure 6:**
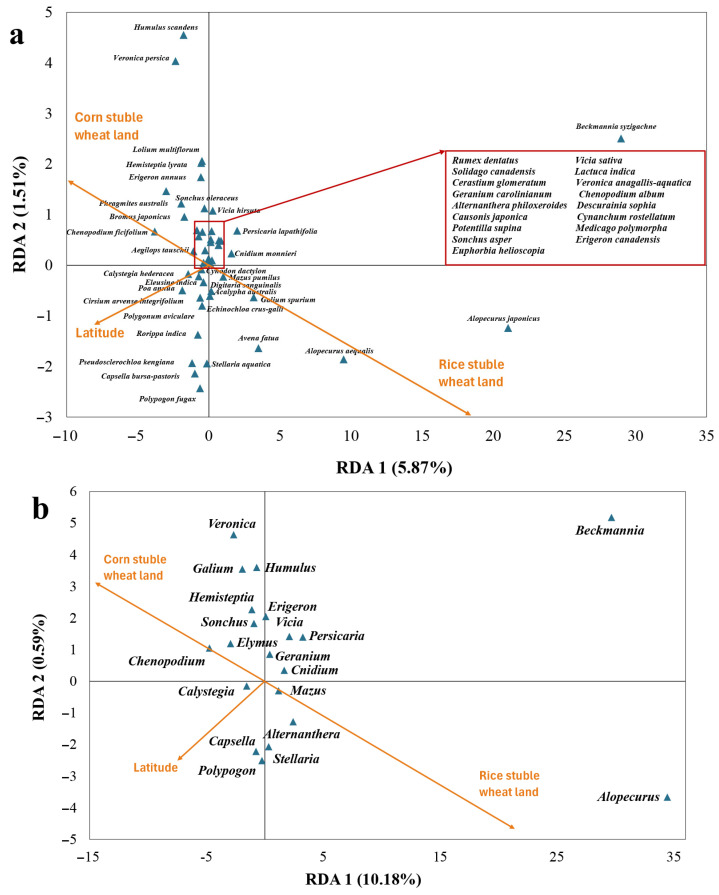
Redundancy analysis (RDA) showing influences of rice or corn stubble wheat lands and latitude of sites on the distributions of common weed species (**a**) and plant genera of common weed species (**b**).

Redundancy analysis (RDA) suggested that the distribution of common weeds (with a frequency >10% among sites) was significantly related to the latitude and type of wheat lands surveyed (Figure 6a). In particular, latitude was positively correlated with corn stubble wheat lands and negatively correlated with rice stubble wheat lands. *Veronica persica*, *Humulus scandens*, *Chenopodium ficifolium*, *Bromus japonicus* Thunb., *Phragmites australis* (Cav.) Trin. ex Steud., *Elymus kamoji*, *Erigeron annuus* (L.) Pers., and *Hemisteptia lyrata* (Bunge) Fisch. & C. A. Mey. were more likely to occur on corn stubble wheat lands, while *Alopecurus japonicus*, *Alopecurus aequalis*, *Avena fatua* L., *Galium spurium*, *Acalypha australis* L., and *Beckmannia syzigachne* were more likely to occur on rice stubble wheat fields. *Polypogon fugax* Nees ex Steud., *Capsella bursa-pastoris*, *Pseudosclerochloa kengiana* (Ohwi) Tzvelev, *Rorippa indica* (L.) Hiern, *Cirsium arvense* var. *integrifolium* Wimm. & Grab., and *Poa annua* L. were more likely to occur in northern regions.

RDA at the genus level showed a similar pattern to that at the species level (Figure 6b). Weed species from genus *Chenopodium, Elymus*, *Sonchus*, *Hemisteptia*, *Galium*, *Veronica*, and *Humulus* were more likely to occur on corn stubble wheat lands, and those from *Alopecurus*, *Beckmannia*, *Alternanthera*, *Mazus*, and *Stellaria* were more likely to occur on rice stubble wheat fields. Weed species from the genus *Capsella*, *Calystegia*, and *Polypogon* were more likely to appear in northern regions.

### 3.6. Diversity Index

**Figure 7:**
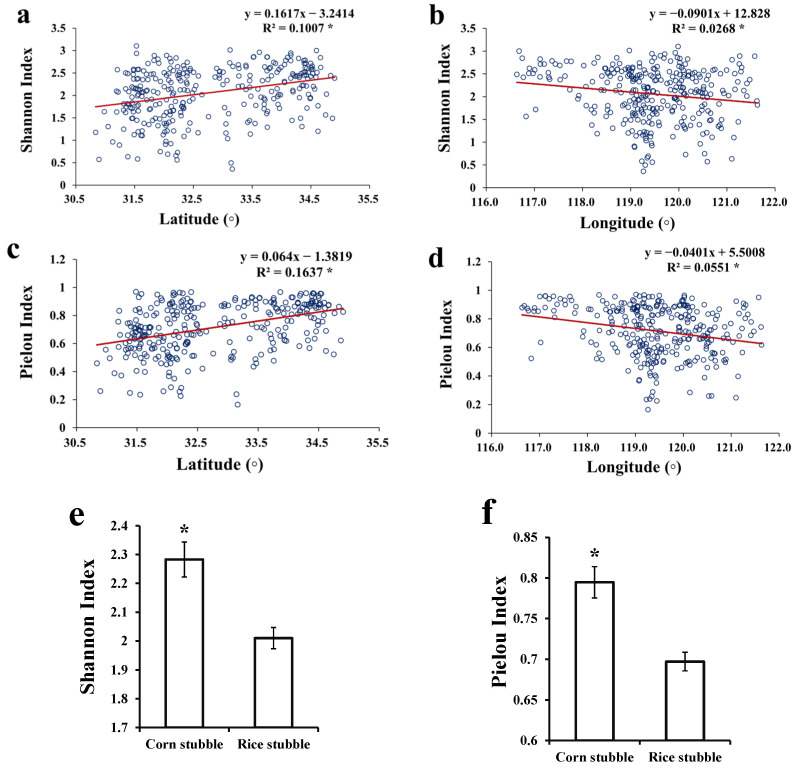
Correlations between Shannon–Wiener α-diversity index (Shannon index, (**a**,**c**)) and Pielou’s evenness index (Pielou index, (**b**,**d**)) of weed communities, latitude or longitude of wheat land sites, and average Shannon α-diversity index (**e**) and Pielou’s evenness index (**f**) of the two types of wheat lands. *: significant correlation at *p* < 0.05.

The Shannon–Wiener α-diversity index and Pielou’s evenness index were both significantly (*p* < 0.05) and positively correlated with the latitude of sites (Figure 7a,c), and significantly and negatively correlated with the longitude (Figure 7b,d). An independent sample *t*-test showed that corn stubble wheat lands had a significantly higher average Shannon–Wiener α-diversity index and Pielou’s evenness index (Figure 7f). 

### 3.7. Distribution and Succession of Survey Sites

**Figure 8:**
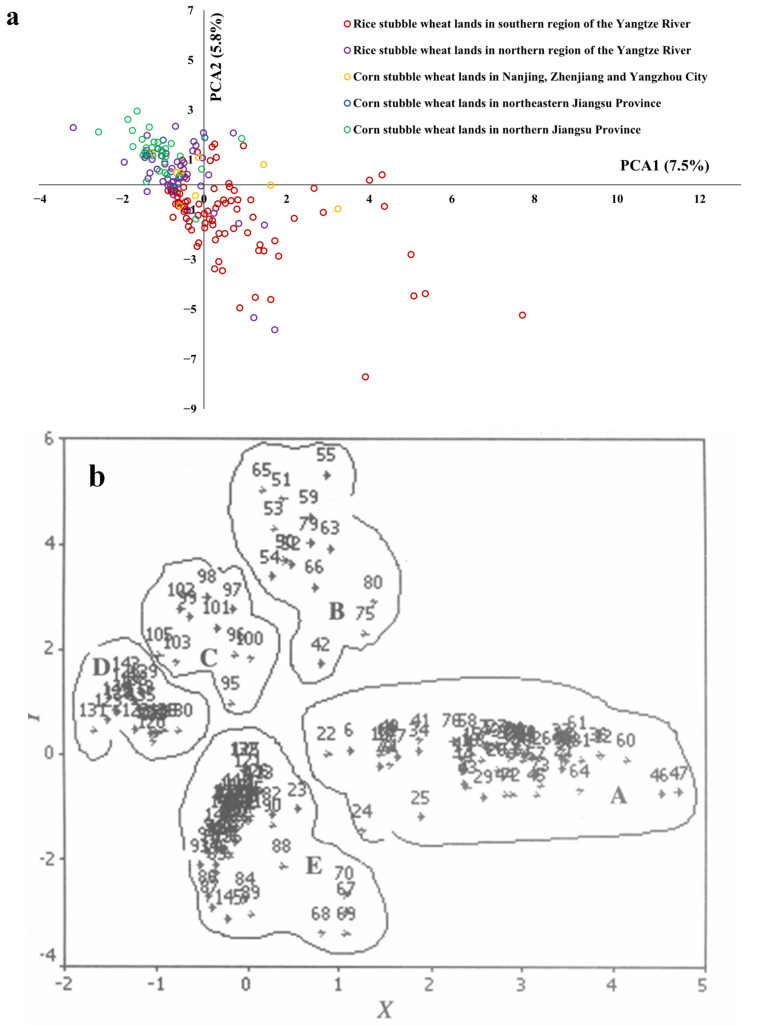
Principal component analysis (PCA) biplots showing the distribution of wheat land sites according to weed communities surveyed in historical data (**a**) cited from Wang and Qiang (2007) and this study (**b**).

In the historical data, wheat land sites can be clearly divided into five groups on the PCA biplot (Figure 8a). In this survey, the PCA results suggested a continuous gradient in the structure of weed communities of wheat lands in Jiangsu Province (Figure 8b), with wheat lands surveyed in northern areas tending to distribute on the top-left and those surveyed in southern areas tending to distribute on the righter parts. Different kinds of wheat land sites mixed on the plot, without clear lines of demarcation.

## 4. Discussion

### 4.1. Large Increase in Wheat Weed Diversity Suggests New Challenges

Wheat weed diversity in the studied area increased significantly with increasing richness in species, genera, and families. Wang and Qiang surveyed 1500 wheat lands from 1999 to 2000 and recorded 117 weed species, belonging to 78 genera and 24 families [13]. Our survey, conducted in 2024 on 924 wheat lands, recorded that weed diversity increased by 33.3% in species, 32.1% in plant genera, and 62.5% in plant families. Several factors contributed to the increase in weed diversity. First, with the urbanization rate in Jiangsu Province increasing from 42.3% in 2000 to 75.5% in 2024 [14], the local wheat-cultivation system has undergone a transition from small-scale and finely managed fields to large-scale and intensive operations with the mechanization of agriculture. With an increasing deficit of labor resources for agricultural work, weed management for wheat lands relies on the application of chemical herbicides in eastern China [21]. Jing et al. (2022) reported that the proportion of herbicides out of total pesticides applied in China increased from 15% in 1999 to 40% in 2018 [22]. Considering the constraints related to wheat seedling injury caused by herbicides, farmers usually undertake one phase of pre-emergence and another phase of post-emergence chemical control practices for one wheat-growing season [21]. Weed plants escaping chemical control on wheat lands are usually not controlled until the wheat harvest, which frequently accumulates large and diverse weed soil seed banks, in particular after the jointing stage of wheat seedlings. The control efficacy of herbicides applied on fields may vary greatly depending on the weather conditions, technologies applied, soil conditions, weed communities on the land patches, and so on. Thus, the performance of herbicide applications frequently varies greatly among lands or among patches of the same lands, which results in highly diverse structures in weed communities, in particular on large farms. Second, full implementation of the “straw returning to the field” policy since 2008 has facilitated weed occurrence on wheat lands, despite providing benefits in many aspects [23]. In the studied areas, arable lands are usually cultivated during two growing seasons for one year. Before 2008, most straw was burned on fields or used for fuel. After implementing the “straw returning to the field” policy, large amounts of straw containing numerous weed seeds were returned to the soil, and continuously accumulated in seed banks. Third, the wide application of minimal tillage before wheat seeding, in lieu of traditional deep plowing, facilitated diverse weed occurrence. Deep plowing at a depth of >20 cm is one of the most frequently used non-chemical control methods against troublesome weeds [24]. However, many farmers, especially those holding big farms, frequently undertake shallow rotary tillage or non-tillage to save on costs and time, carrying out rush seeding before winter. Moreover, optimized tillage and fertilization practices can significantly enhance weed community diversity [11]. In addition, alien plant invasions have also increased weed diversity [4]. During the rapid urbanization over the past 25 years in Jiangsu Province, the construction of traffic networks and urban infrastructure increased dramatically. Many alien invasive weeds spread into wheat-planting areas via various trades, communications, and the movement of agricultural machinery, and became troublesome, such as *Lolium multiflora*, *Aegilops tauschii* and *Alopecurus myosuroides* [25,26,27]. Additionally, various environmental weed species invaded and became common wheat weeds, such as *Chenopodium ficifolium*, *Solidago canadensis*, *Erigeron* spp., *Sonchus* spp., *Cynanchum rostellatum*, *Cnidium monnieri*, and *Humulus scandens*.

Increasing weed diversity caused increasing challenges to weed management on wheat lands. Weed control spectra of a certain wheat herbicide are limited, and different weed species may have different sensitivities to various herbicides. Developing a new herbicide is very costly and usually takes >10 years [28]. Thus, increasing weed diversity requires increasing chemical herbicides, which is a significant challenge. Moreover, different weed species hold different adaptive traits and strategies to environmental conditions. Increasing weed diversity has also posed increasing challenges for integrated weed management strategies. For example, several invasive weed species from the Poaceae family frequently cause serious yield losses of wheat in China, including *Aegilops tauschii* [26,29], *Alopecurus myosuroides* [30,31], and *Lolium multiflorum* [25]. *Aegilops tauschii* is one of the homologs of wheat and is very difficult to control by wheat herbicides [21]. It is usually recommended that wheat lands infested by *Aegilops tauschii* undergo rice–wheat rotation or use plant broadleaf crops in lieu of wheat, or undergo deep plowing every year [21]. Moreover, herbicide-resistant *Aegilops tauschii* [26,29], *Alopecurus myosuroides* [30,31], and *Lolium multiflorum* [25] have been reported in wheat lands in China.

### 4.2. Grassy Weeds Have Become Even More Dominant

Compared with 25 years ago, grassy weeds increased in both the proportions of species richness and dominance values out of the overall weed species. Specifically, the number of grassy weed species increased from 14 to 23, a 64.3% increase. More importantly, the proportion of the dominance value of grassy weeds out of the overall weeds increased from 40.4% in the historical survey [13] to 54.3% in this study. The increasing seriousness of grassy weeds is primarily due to the increasing reliance on chemical control against weeds. Grassy weeds and wheat belong to the same plant family, and share similar growing traits and physiology; in particular, they share similar sensitivities to various herbicides. Therefore, wheat herbicides targeting grassy weeds usually present high risks of causing wheat seedling injury, which introduces many limits in chemical control against grassy weeds on wheat lands [21]. Moreover, growing points of grassy weed seedlings are enclosed within multiple layers of leaf sheaths, while herbicides need to be absorbed and transported in seedlings to kill growing points. Considering that many troublesome grassy weed species hold powerful tillering and re-growing abilities, chemical control practices may fail, as long as one growing point of a weed plant survives [32,33,34]. Moreover, grassy weeds surviving chemical control may generate herbicide-resistant seeds and accumulate soil seed banks before crop harvest [33,35]. Therefore, grassy weeds on wheat lands represent a group of the most troublesome weeds for wheat and other cereal crops such as rice, corn, and sorghum [21,36].

*Beckmannia syzigachne* and three *Alopecurus* weed species were dominant wheat weeds in eastern China. *Beckmannia syzigachne* has become the most troublesome weed species, particularly on rice stubble wheat lands. The proportion in dominance value of *Beckmannia syzigachne* out of the overall weeds in this study was as high as 19.2%, while it was 6.6% in the historical survey conducted 25 years earlier [13]. *Beckmannia syzigachne* has a vigorous tillering capacity and heightens competition against wheat seedlings [37,38]. Moreover, herbicide-resistant *Beckmannia syzigachne* is frequently reported in eastern China [34]. *Alopecurus* spp., including *Alopecurus aequalis*, *Alopecurus japonicus*, and *Alopecurus myosuroides*, occupied 16.2% dominance values out of the overall weeds, compared to 15.3% in the historical survey results. *Alopecurus aequalis* and *Alopecurus japonicus* are both traditional serious weeds species in wheat lands in eastern China [39]. Meanwhile, *Alopecurus myosuroides* was not recorded in the historical survey of 1500 wheat lands in Jiangsu. Herbicide resistance in the three *Alopecurus* species has also been reported in eastern China, in particular in *Alopecurus japonicus* [40,41]. The dominance value of *Alopecurus japonicus* was second only to *Beckmannia syzigachne* in this survey, and the third most dominant weed species in the historical data (preceded only by *Veronica persica* and *Galium spurium*). *Alopecurus myosuroides* is increasingly troublesome and quickly distributing in many wheat-planting areas in China, with a quick evolution of herbicide resistance [42,43,44].

Several grassy weed species became new dominant weed species on wheat lands, having not been recorded in the historical data, such as *Lolium multiflorum*, *Aegilops tauschii*, and *Bromus japonicus. Lolium multiflorum* is a serious wheat weed species in Europe, Australia, North America, and centra China [45]. *Aegilops tauschii* and *Bromus japonicus* frequently infest wheat lands with corn stubble, and their seed banks could be greatly depleted in rice–wheat rotation systems [46]. Furthermore, nine other grassy weed species were recorded in the data from this study, which were not identified in the historical data.

In contrast, several low-growing grassy weed species became less dominant compared with historical survey in the studied area, such as *Pseudosclerochloa kengiana*, *Polypogon fugax*, *Avena fatua*, and *Alopecurus aequalis*. In the past 25 years, the average wheat yield increased from 4,755 kg/ha. to 5,748 kg/ha. Wheat plants have become much denser and more robust on fields compared to 25 years ago, which usually presents great competitive advantages against weeds after the jointing stage. Thus, low-growing grassy weeds are usually suppressed by wheat plants after the jointing stage. Low-growing grassy weeds are likely to continuously decrease in dominance with the increasing application of high-yield and high-efficiency wheat-cultivation strategies [47].

### 4.3. Several Broadleaf Weeds Occasionally Cause Serious Challenges

The overall dominance values of most broadleaf weeds decreased considerably compared to the historical data, such as weeds of *Galium* spp., *Vicia* spp., and *Veronica* spp. Broadleaf weeds could be more easily controlled by wheat herbicides, as their growing points are usually bare, and, more importantly, relatively distant from wheat in the phylogenetic tree [21]. Many wheat herbicides with very low risks to wheat seedlings could be used for broadleaf weed control, such as florasulam, fluroxypyr, halauxifen, carfentrazone, and tribenuron [5]. Meanwhile, some broadleaf weed species remain troublesome and occasionally cause serious yield losses, such as *Galium spurium*, *Stellaria* spp., *Capsella bursa-pastoris, Geranium* spp., *Veronica* spp., and *Chenopodium* spp. Moreover, the dominance values of some broadleaf weeds increased over the past 25 years, such as *Geranium carolinianum*, *Humulus scandens*, and *Capsella bursa-pastoris*, which might be due to adaptations to chemical control strategies and quickly growing from ridges to the canopy of wheat plants. *Geranium carolinianum* seedlings are usually robust, with a rosette growing form, and are frequently covered by wheat or other weed plants. Consequently, a portion of *Geranium carolinianum* seedlings frequently receive lower doses of herbicides applied during winter or early spring; moreover, this weed species is not sensitive to several wheat herbicides designed to target broadleaf weeds [21,48]. *Capsella bursa-pastoris* was one of the most troublesome herbicide-resistant weed species in wheat lands in China, and this weed species frequently becomes insensitive to various wheat herbicides after the bolting stage [21]. *Humulus scandens* is a large herbaceous vine plant, which is frequently distributed on ridges around wheat lands and spreads to wheat lands by quickly growing in warm conditions. In the past, most farmers held small farms with limited land, and they commonly controlled weeds on ridges through manual removal or other methods. In modern agricultural areas with very limited labor forces and much more land, most farmers do not manage weeds on ridges as they did before. Furthermore, some alien invasive plant species have invaded wheat lands and become common weed species in the studied areas, such as *Geranium dissectum*, *Medicago* spp., and several species from Compositae. *Geranium dissectum* was first reported in Jiangsu Province, China, in 2013 and has since invaded oilseed rape and wheat fields [4,49,50].

### 4.4. Homogenization in Wheat Weed Communities

A clear trend of weed community homogenization among different areas and types of wheat lands was observed. Biotic homogenization is a process by which the genetic, taxonomic, or functional similarities of regional biotas increase over time [51], which may be propelled by the combined effects of climatic change, increasing communications at different scales, and farming practices [52,53,54,55]. This process reduces functional diversity, thereby weakening essential ecological functions and making the system more vulnerable to environmental stress and biological invasions. The surveyed area spans from 30°46′ to 35°07′ N and 116°22′ to 121°55′ E, with both latitudinal and longitudinal ranges exceeding 4°. Within this range, both annual mean temperature and precipitation decrease significantly from southeast to northwest. As a result, farmlands in the northwestern region are generally more arid, with more wheat lands undergoing a corn–wheat rotation, in lieu of rice–wheat rotation. In contrast, the southeastern and central regions commonly adopt a rice–wheat rotation. During the rice-growing season (June to October), fields are frequently kept moist or flooded, which continuously depletes weed soil seed banks ready to germinate in moist soil conditions. On the other hand, during the growing seasons of corn or other highland crops from June to October, the drier conditions limit the seed germination of many weeds and thus effectively preserve soil seed banks. In this survey, corn stubble wheat lands showed significantly higher weed diversity than rice stubble wheat lands; and weed diversity significantly correlated with latitude and longitude. Nevertheless, discriminations in weed communities between corn and rice stubble wheat lands, as well as among different areas, decreased significantly compared to the historical data.

Homogenization in wheat weed communities in the studied areas should be attributed firstly to the development of wheat cultivation across the whole province. Over the past 25 years, updated wheat varieties and standardized cultivating technologies have been promoted throughout the province, powerfully propelled by the government. Many more arable lands adopted rice–wheat rotations, which propelled homogenization in wheat weed communities. Mechanization in planting, tillage, pesticide application, and harvesting has replaced traditional manual methods in most arable lands in Jiangsu Province over the past 25 years [56]. Wheat weed communities across different wheat lands tended to be more similar when subjected to similar selection pressures from the same cultivating systems. Moreover, seeds of various troublesome weeds were distributed by agricultural machinery [57] or wheat seed contamination [58].

Second, alien plant invasions also propelled homogenization in wheat weed communities, as above discussed [59]. The number of alien invasive weed species has increased from 30 in historical records to 42 at present, with many becoming newly dominant species. Similar patterns of weed community homogenization have also been observed in other regions of eastern China. For example, in Anhui Province, long-term surveys of summer crop fields revealed increased dominance of alien species and co-dominance patterns replacing formerly single dominant weeds [60]. Moreover, climate change may further accelerate the homogenization of biological communities through mechanisms such as rising temperatures, altered precipitation patterns, and extended growing seasons [61]. A paired-sample *t*-test indicated no significant difference in the average temperatures of the 13 cities surveyed during the wheat-growing season (November to May) between 1999 and 2024 (*p* = 0.12), suggesting limited climatic change over the past 25 years [14]. In addition, Jiangsu Province spans from subtropical to temperate climatic zones, with higher annual precipitation, and a well-developed irrigation system. The influences of climate changes on wheat weed communities in Jiangsu over the past two decades mainly stemmed from extreme temperature fluctuations, which facilitated wheat seedling injury, and drove farmers to improve upon chemical control strategies with herbicides that provide greater safety for wheat seedlings. Furthermore, long-term monitoring data show that soil nutrient levels in rice fields across Jiangsu have generally increased, particularly in nitrogen, organic matter, and potassium contents, and that soil pH has gradually declined, indicating a trend toward acidification [62]. These shifts in soil nutrient composition may have also influenced the succession of wheat weed communities.

## 5. Conclusions

This study revealed the successional dynamics of weed communities in wheat fields across Jiangsu Province over the past 25 years, against a background of transition in agricultural production from traditional intensive farming to large-scale, mechanized, and intensified systems. Both weed species richness and community diversity increased greatly. The species richness and dominance value of grassy weeds have risen markedly, while the dominance of broadleaf weeds has decreased. *Beckmannia syzigachne*, *Alopecurus japonicus*, *Alopecurus myosuroides, Lolium multiflorum*, *Aegilops tauschii*, and *Bromus japonicus* were the main dominant weed species in wheat lands of Jiangsu Province, while several low-growing grassy weed species became less dominant, such as *Pseudosclerochloa kengiana*, *Polypogon fugax*, *Avena fatua*, and *Alopecurus aequalis*. A clear trend of weed community homogenization among different areas and types of wheat lands was observed. Additionally, grassy weeds dominated the rice stubble wheat fields in central and southeastern regions, whereas broadleaf weeds were more prevalent in the corn stubble wheat lands of the northwestern region. For wheat-planting areas subject to urbanization, agricultural intensification, and reliance on chemical control, increasing weed diversity is probably inevitable. Integrated weed management should be highlighted, such as strengthening field management, carrying out deep plowing every year, improving straw management and crop rotation strategies, and plant quarantine to block plant invasions. Grassy weeds remain be the most problematic weeds, in particular for robust grassy weeds and those readily evolving herbicide resistances. Moreover, it is important to block and intercept new invasions of invasive grassy weed species in wheat-planting areas. Broadleaf weeds are generally easier to control with herbicides, while different broadleaf weed species require different control strategies. For example, *Capsella bursa-pastoris*, with tiny seeds, is sensitive to many pre-emergence wheat herbicides and to various post-emergence wheat herbicides before the heading stage, while it is highly tolerant to post-emergence wheat herbicides after the bolting stage [21]. *Galium spurium*, with large seeds, is not sensitive to several pre-emergence herbicides, while it could be controlled by halauxifen and fluroxypyr even at the bolting stage [21]. Against a background of weed community homogenization among different areas and types of wheat lands, communicating and promoting precise and highly efficient weed management technologies could be even more important. In addition, field surveys should also be highlighted to monitor weed community succession and adjust weed managing strategies accordingly. This study provides the first systematic investigation of weed community succession in wheat fields against the background of a shift in wheat-cultivation systems from traditional to modern methods undertaken at a large scale. The insights revealed here could be important for improving weed management strategies in different areas and for understanding the evolution of weed communities on arable lands. It is worth noting that limitations may exist when making comparisons with historical data, such as non-overlapping survey sites between historical and current studies, and variations in sampling densities. These limitations may be improved in future studies. 

## Figures and Tables

**Table 1:** Visual scoring method for weed dominance values according to average coverage values of weed species on the sampled field.

Code	Average Height on Sampled Land		
	>80cm	20–80cm	<20cm
0.1	<0.1%	<1%	<2%
0.5	0.2–0.9%	1–2%	3–5%
1	1–2%	3–5%	6–10%
2	3–5%	6–10%	11–25%
3	6–10%	11–25%	25–50%
4	11–25%	25–50%	50–90%
5	>25%	>50%	>90%

**Table 2:** Frequency and average dominance values of common weed species (frequence > 10% among sites surveyed) among 308 wheat land sites surveyed in 2024 (Fr-C and Dv-C), and those among 150 wheat land sites surveyed in 1999–2000 (Fr-H and Dv-H).

Code	Family	Species	Fr-C (%)	Fr-H (%)	Dv-C (%)	Dv-H (%)
1	Geraniaceae	*Geranium carolinianum*	86.4	22.4	3	2
2	Rubiaceae	*Galium spurium*	76	57.1	7.4	14.7
3	Poaceae	*Beckmannia syzigachne*	74.7	22.4	19.2	6.6
4	Plantaginaceae	*Veronica persica*	64.6	50.9	3.3	11.7
5	Cannabaceae	*Humulus scandens*	53.6	\	2.8	\
6	Poaceae	*Elymus kamoji*	52.9	\	1.6	\
7	Brassicaceae	*Capsella bursa-pastoris*	52.3	11.1	1.9	1
8	Compositae	*Erigeron canadensis*	51.3	\	1	\
9	Convolvulaceae	*Calystegia hederacea*	51	11.8	1.8	0.8
10	Amaranthaceae	*Alternanthera philoxeroides*	49.4	3	2	0
11	Poaceae	*Alopecurus japonicus*	49.4	31.8	9.4	8.6
12	Fabaceae	*Vicia sativa*	47.7	46.4	1.3	5.7
13	Compositae	*Hemisteptia lyrata*	44.8	8.5	1.3	0.3
14	Mazaceae	*Mazus pumilus*	43.2	5.4	1	0.2
15	Amaranthaceae	*Chenopodium ficifolium*	41.6	\	1.6	\
16	Poaceae	*Polypogon fugax*	41.6	26.1	1.8	3.9
17	Caryophyllaceae	*Stellaria aquatica*	39.3	10.6	1.9	1.4
18	Apiaceae	*Cnidium monnieri*	38.6	\	1.1	\
19	Poaceae	*Alopecurus aequalis*	38.3	27.5	5	6.7
20	Polygonaceae	*Persicaria lapathifolia*	36.4	\	1.4	\
21	Poaceae	*Lolium multiflorum*	34.1	\	6.5	\
22	Apocynaceae	*Cynanchum rostellatum*	31.5	\	0.8	\
23	Euphorbiaceae	*Acalypha australis*	31.2	\	0.5	\
24	Poaceae	*Poa annua*	29.2	8.6	0.9	0.9
25	Compositae	*Cirsium arvense*	28.6	20.5	0.7	1.9
26	Polygonaceae	*Rumex dentatus*	27.3	4.3	0.5	0.2
27	Euphorbiaceae	*Euphorbia helioscopia*	26.9	11	0.3	1
28	Poaceae	*Bromus japonicus*	26	\	1.2	\
29	Polygonaceae	*Polygonum aviculare*	22.7	9.5	0.8	0.5
30	Plantaginaceae	*Veronica anagallis-aquatica*	22.4	3.5	0.5	0.1
31	Poaceae	*Phragmites australis*	22.4	\	1	\
32	Poaceae	*Digitaria sanguinalis*	22.1	\	0.4	\
33	Compositae	*Sonchus oleraceus*	21.8	\	0.5	\
34	Poaceae	*Avena fatua*	21.1	28.6	1.5	5.8
35	Compositae	*Lactuca indica*	20.1	\	0.3	\
36	Compositae	*Sonchus asper*	19.5	\	0.3	\
37	Poaceae	*Cynodon dactylon*	19.2	\	0.3	\
38	Fabaceae	*Medicago polymorpha*	16.9	\	0.4	\
39	Fabaceae	*Vicia hirsuta*	14.9	\	0.7	\
40	Poaceae	*Eleusine indica*	14.6	\	0.3	\
41	Poaceae	*Echinochloa crus-galli*	14.3	\	0.4	\
42	Brassicaceae	*Rorippa indica*	13.6	4	0.6	0.3
43	Compositae	*Erigeron annuus*	13	\	0.3	\
44	Compositae	*Solidago canadensis*	12.7	\	0.4	\
45	Brassicaceae	*Descurainia sophia*	11.7	19.8	0.2	3.6
46	Amaranthaceae	*Chenopodium album*	11.4	7.7	0.4	0.7
47	Poaceae	*Aegilops tauschii*	10.7	\	0.5	\
48	Vitaceae	*Causonis japonica*	10.7	\	0.2	\
49	Caryophyllaceae	*Cerastium glomeratum*	10.4	22.7	0.2	3.1
50	Rosaceae	*Potentilla supina*	10.4	\	0.2	\

Note that the data for all surveyed weed species are provided in Table S1.

**Table 3:** Mean dominance values (×1000) of common weed species among corn stubble and rice stubble wheat fields.

Species	Corn Stubble	Rice Stubble
*Veronica persica* *	63.2 ± 20.5	20.5 ± 2.8
*Echinochloa crus-galli* *	0.2 ± 0.2	4.2 ± 0.9
*Polypogon fugax* *	4.8 ± 1.3	17.9 ± 3.2
*Descurainia sophia* *	4.5 ± 0.7	0.8 ± 0.4
*Capsella bursa-pastoris* *	8.8 ± 2.1	18.0 ± 3.1
*Humulus scandens* *	74.5 ± 22.9	12.9 ± 2.1
*Stellaria aquatica* *	6.6 ± 4.1	18.6 ± 3.3
*Alopecurus japonicus* *	18.4 ± 12.2	94.8 ± 14.8
*Acalypha australis* *	2.1 ± 0.6	4.9 ± 0.6
*Mazus pumilus* *	2.3 ± 1.3	9.8 ± 1.0
*Beckmannia syzigachne* *	51.8 ± 21.7	189.2 ± 16.6
*Galium spurium* *	39.6 ± 8.6	68.9 ± 9.2
*Veronica anagallis-aquatica*	6.8 ± 4.4	4.1 ± 0.7
*Polygonum aviculare*	5.0 ± 2.5	7.5 ± 2.0
*Potentilla supina*	2.0 ± 1.0	1.4 ± 0.4
*Rumex dentatus*	3.9 ± 1.0	4.2 ± 1.0
*Lactuca indica*	4.3 ± 1.1	2.8 ± 0.8
*Cirsium arvense*	9.6 ± 5.9	5.5 ± 1.6
*Calystegia hederacea*	21.6 ± 7.3	14.4 ± 2.2
*Vicia sativa*	8.2 ± 2.2	12.6 ± 3.6
*Lolium multiflorum*	59.3 ± 17.6	55.1 ± 10.7
*Elymus kamoji*	30.5 ± 14.6	9.4 ± 1.7
*Cynodon dactylon*	5.0 ± 1.4	2.5 ± 0.6
*Rorippa indica*	1.1 ± 0.4	5.7 ± 2.3
*Solidago canadensis*	3.2 ± 1.4	3.6 ± 1.6
*Aegilops tauschii*	10.4 ± 2.7	3.0 ± 1.7
*Alopecurus aequalis*	21.4 ± 13.8	47.6 ± 7.7
*Alternanthera philoxeroides*	37.5 ± 16.5	12.3 ± 1.7
*Sonchus oleraceus*	12.0 ± 7.2	3.2 ± 0.9
*Chenopodium album*	11.6 ± 10.2	1.9 ± 0.5
*Phragmites australis*	24.3 ± 12.2	5.1 ± 1.3
*Cynanchum rostellatum*	16.6 ± 13.0	5.3 ± 1.2
*Digitaria sanguinalis*	4.3 ± 1.2	3.6 ± 0.6
*Medicago polymorpha*	6.3 ± 4.8	2.7 ± 0.5
*Hemisteptia lyrata*	23.8 ± 8.2	8.2 ± 1.6
*Eleusine indica*	5.2 ± 2.0	2.2 ± 0.9
*Cerastium glomeratum*	1.3 ± 0.6	2.1 ± 0.6
*Bromus japonicus*	24.5 ± 12.1	7.0 ± 2.3
*Cnidium monnieri*	2.9 ± 0.8	10.8 ± 2.7
*Persicaria lapathifolia*	7.0 ± 3.9	13.8 ± 2.8
*Causonis japonica*	2.5 ± 0.7	1.5 ± 0.4
*Vicia hirsuta*	7.0 ± 3.8	5.8 ± 2.8
*Erigeron canadensis*	7.9 ± 1.3	9.2 ± 1.4
*Chenopodium ficifolium*	33.9 ± 17.3	9.6 ± 1.7
*Sonchus asper*	7.9 ± 4.2	2.3 ± 0.4
*Geranium carolinianum*	24.6 ± 3.8	25.9 ± 2.7
*Avena fatua*	19.6 ± 7.8	11.6 ± 5.3
*Erigeron annuus*	10.9 ± 8.4	1.1 ± 0.2
*Poa annua*	13.4 ± 8.5	6.6 ± 1.1
*Euphorbia helioscopia*	4.6 ± 1.1	2.8 ± 0.3

Note: Values are mean ± SEs. *: significant difference between corn stubble and rice stubble fields at *p* < 0.05 (independent *t*-test).

## Data Availability

The data presented in this study are available upon request from the corresponding author due to privacy restrictions.

## Supplementary Materials

The following supporting information can be downloaded at: https://www.mdpi.com/article/10.3390/biology14080943/s1, **Table S1:** Family, frequency among 308 sites of rice fields surveyed in 2024 (Fr-C), frequency among 150 sites of rice fields surveyed in 1999-2000 (Fr-H), proportion in dominance value out of overall weeds surveyed in 2024 (Dv-C) and proportion in dominance value out of overall weeds surveyed in 1999-2000 (Dv-H) of weed species. **Table S2:** Proportions in dominance values and species richness out of overall weeds for different plant families. **Table S3:** Proportions in dominance values and species richness out of overall weeds for different plant genera.
