# Supplementary material for: Succession of Weed Community on Wheat Lands in the Past 25 Years: A Case Study in Eastern China
Source: Biology (Basel). 2025 Jul 27;14(8):943. doi: 10.3390/biology14080943 (PMC12383366; doi:10.3390/biology14080943)
Supplement: Supplementary file 1 [file biology-14-00943-s001.zip › biology-3752418-supplementary.pdf]

Table S1. Family, frequency among 308 sites of rice fields surveyed in 2024 (Fr-C), frequency among 150 sites of rice fields surveyed in 1999-2000 (Fr-H), proportion in dominance value out of overall weeds surveyed in 2024 (Dv-C) and proportion in dominance value out of overall weeds surveyed in 1999-2000 (Dv-H) of weed species.

Code	Family	Species	Fr-C (%)	Fr-H (%)	Dv-C (%)	Dv-H (%)
1	Acanthaceae	*Strobilanthes japonica*	0.3	4.4	0.0	0.8
2	Acoraceae	*Acorus calamus*	0.3	\	0.0	\
3	Amaranthaceae	*Oxybasis glauca*	0.3	\	0.0	\
4	Amaranthaceae	*Chenopodium ficifolium*	41.6	\	1.6	\
5	Amaranthaceae	*Chenopodium album*	11.4	7.7	0.4	0.7
6	Amaranthaceae	*Alternanthera philoxeroides*	49.4	3.0	2.0	0.0
7	Amaranthaceae	*Amaranthus viridis*	4.5	\	0.1	\
8	Amaranthaceae	*Amaranthus retroflexus*	1.3	\	0.0	\
9	Amaranthaceae	*Amaranthus blitum*	0.3	\	0.0	\
10	Apiaceae	*Daucus carota*	1.0	\	0.0	\
11	Apiaceae	*Torilis scabra*	1.0	\	0.0	\
12	Apiaceae	*Cnidium monnieri*	38.6	\	1.1	\
13	Apiaceae	*Oenanthe javanica*	0.3	\	0.0	\
14	Apocynaceae	*Cynanchum rostellatum*	31.5	\	0.8	\
15	Araceae	*Pinellia ternata*	1.0	\	0.0	\
16	Boraginaceae	*Bothriospermum zeylanicum*	4.5	3.5	0.1	0.1
17	Boraginaceae	*Bothriospermum chinense*	0.3	\	0.0	\
18	Boraginaceae	*Trigonotis peduncularis*	6.2	3.7	0.1	0.2
19	Boraginaceae	*Lithospermum arvense*	1.0	15.2	0.0	3.1
20	Brassicaceae	*Descurainia sophia*	11.7	19.8	0.2	3.6
21	Brassicaceae	*Lepidium didymum*	1.6	\	0.1	\
22	Brassicaceae	*Rorippa indica*	13.6	4.0	0.6	0.3
23	Brassicaceae	*Rorippa globosa*	0.6	\	0.0	\
24	Brassicaceae	*Rorippa cantoniensis*	0.6	\	0.0	\
25	Brassicaceae	*Capsella bursa-pastoris*	52.3	11.1	1.9	1.0
26	Brassicaceae	*Cardamine flexuosa*	1.9	\	0.0	\

27	Brassicaceae	*Cardamine occulta*	1.0	\	0.0	\
28	Campanulaceae	*Lobelia chinensis*	1.6	\	0.0	\
29	Cannabaceae	*Humulus scandens*	53.6	\	2.8	\
30	Caryophyllaceae	*Stellaria aquatica*	39.3	10.6	1.9	1.4
31	Caryophyllaceae	*Stellaria pallida*	1.3	\	0.0	\
32	Caryophyllaceae	*Stellaria media*	1.0	\	0.0	\
33	Caryophyllaceae	*Cerastium glomeratum*	10.4	22.7	0.2	3.1
34	Caryophyllaceae	*Sagina japonica*	1.6	\	0.1	\
35	Caryophyllaceae	*Arenaria serpyllifolia*	0.3	\	0.0	\
36	Compositae	*Lapsanastrum apogonoides*	2.6	13.2	0.1	2.1
37	Compositae	*Carduus nutans*	0.6	\	0.0	\
38	Compositae	*Erigeron canadensis*	51.3	\	1.0	\
39	Compositae	*Erigeron annuus*	13.0	\	0.3	\
40	Compositae	*Erigeron philadelphicus*	4.5	\	0.1	\
41	Compositae	*Erigeron sumatrensis*	2.6	\	0.0	\
42	Compositae	*Erigeron bonariensis*	0.3	16.6	0.0	0.9
43	Compositae	*Bidens frondosa*	4.9	\	0.1	\
44	Compositae	*Bidens pilosa*	2.9	\	0.0	\
45	Compositae	*Artemisia annua*	1.0	\	0.0	\
46	Compositae	*Artemisia argyi*	0.3	\	0.0	\
47	Compositae	*Artemisia lavandulifolia*	0.3	\	0.0	\
48	Compositae	*Youngia japonica*	1.3	\	0.0	\
49	Compositae	*Youngia erythrocarpa*	0.3	\	0.0	\
50	Compositae	*Cirsium arvense*	28.6	20.5	0.7	1.9
51	Compositae	*Cirsium japonicum*	0.3	\	0.0	\
52	Compositae	*Sonchus oleraceus*	21.8	\	0.5	\
53	Compositae	*Sonchus asper*	19.5	\	0.3	\
54	Compositae	*Ixeris polycephala*	6.2	11.8	0.1	0.5
55	Compositae	*Eclipta prostrata*	2.3	\	0.0	\

56	Compositae	*Symphyotrichum subulatum*	5.8	\	0.1	\
57	Compositae	*Hemisteptia lyrata*	44.8	8.5	1.3	0.3
58	Compositae	*Pseudognaphalium affine*	6.8	2.9	0.1	0.1
59	Compositae	*Lactuca indica*	20.1	\	0.3	\
60	Compositae	*Helianthus tuberosus*	0.3	\	0.0	\
61	Compositae	*Solidago canadensis*	12.7	\	0.4	\
62	Convolvulaceae	*Calystegia hederacea*	51.0	11.8	1.8	0.8
63	Convolvulaceae	*Ipomoea nil*	0.3	\	0.0	\
64	Convolvulaceae	*Ipomoea purpurea*	1.0	\	0.0	\
65	Convolvulaceae	*Ipomoea hederacea Jacq.*	0.3	\	0.0	\
66	Cyperaceae	*Bolboschoenus planiculmis*	2.3	\	0.2	\
67	Cyperaceae	*Cyperus serotinus*	2.6	\	0.0	\
68	Cyperaceae	*Cyperus rotundus*	2.3	\	0.1	\
69	Cyperaceae	*Cyperus difformis*	0.3	\	0.0	\
70	Equisetaceae	*Equisetum hyemale*	5.8	\	0.1	\
71	Euphorbiaceae	*Euphorbia helioscopia*	26.9	11.0	0.3	1.0
72	Euphorbiaceae	*Euphorbia prostrata*	1.0	\	0.0	\
73	Euphorbiaceae	*Euphorbia maculata*	0.3	\	0.0	\
74	Euphorbiaceae	*Acalypha australis*	31.2	\	0.5	\
75	Fabaceae	*Melilotus suaveolens*	0.6	\	0.0	\
76	Fabaceae	*Trifolium repens*	1.0	\	0.0	\
77	Fabaceae	*Glycine soja*	3.9	\	0.1	\
78	Fabaceae	*Aeschynomene indica*	7.5	\	0.1	\
79	Fabaceae	*Medicago polymorpha*	16.9	\	0.4	\
80	Fabaceae	*Medicago minima*	0.3	\	0.0	\
81	Fabaceae	*Vicia sativa*	47.7	46.4	1.3	5.7
82	Fabaceae	*Vicia hirsuta*	14.9	\	0.7	\
83	Fabaceae	*Vicia cracca*	9.1	4.0	0.3	0.1

84	Fabaceae	*Vicia sativa nigra*	2.6	\	0.0	\
85	Fabaceae	*Vicia tetrasperma*	0.3	\	0.0	\
86	Geraniaceae	*Geranium carolinianum*	86.4	22.4	3.0	2.0
87	Geraniaceae	*Geranium dissectum*	1.0	\	0.1	\
88	Lamiaceae	*Salvia plebeia*	6.2	3.5	0.1	0.2
89	Lamiaceae	*Lagopsis supina*	0.6	\	0.0	\
90	Lamiaceae	*Lamium amplexicaule*	1.0	\	0.0	\
91	Lamiaceae	*Leonurus japonicus*	2.9	\	0.1	\
92	Lythraceae	*Ammannia baccifera*	0.3	\	0.0	\
93	Malvaceae	*Abutilon theophrasti*	3.2	\	0.0	\
94	Mazaceae	*Mazus pumilus*	43.2	5.4	1.0	0.2
95	Moraceae	*Broussonetia papyrifera*	0.3	\	0.0	\
96	Onagraceae	*Ludwigia prostrata*	0.3	\	0.0	\
97	Oxalidaceae	*Oxalis corniculata*	1.6	\	0.0	\
98	Oxalidaceae	*Oxalis pes-caprae*	0.3	\	0.0	\
99	Papaveraceae	*Papaver rhoeas*	0.3	\	0.0	\
100	Phytolaccaceae	*Phytolacca americana*	1.3	\	0.0	\
101	Plantaginaceae	*Plantago asiatica*	1.0	3.6	0.0	0.3
102	Plantaginaceae	*Plantago virginica*	0.3	\	0.0	\
103	Plantaginaceae	*Veronica persica*	64.6	50.9	3.3	11.7
104	Plantaginaceae	*Veronica anagallis-aquatica*	22.4	3.5	0.5	0.1
105	Plantaginaceae	*Veronica peregrina*	9.4	\	0.1	\
106	Plantaginaceae	*Veronica arvensis*	6.8	\	0.1	\
107	Plantaginaceae	*Veronica polita*	0.3	\	0.0	\
108	Poaceae	*Echinochloa crus-galli*	14.3	\	0.4	\
109	Poaceae	*Polypogon fugax*	41.6	26.1	1.8	3.9
110	Poaceae	*Setaria viridis*	6.8	\	0.1	\
111	Poaceae	*Setaria faberi*	1.3	\	0.0	\
112	Poaceae	*Cynodon dactylon*	19.2	\	0.3	\
113	Poaceae	*Lolium multiflorum*	34.1	\	6.5	\

114	Poaceae	*Lolium perenne*	2.3	\	0.1	\
115	Poaceae	*Leersia japonica*	8.8	\	1.4	\
116	Poaceae	*Pseudosclerochloa kengiana*	3.6	23.4	0.5	7.2
117	Poaceae	*Alopecurus japonicus*	49.4	31.8	9.4	8.6
118	Poaceae	*Alopecurus aequalis*	38.3	27.5	5.0	6.7
119	Poaceae	*Alopecurus myosuroides*	8.1	\	1.8	\
120	Poaceae	*Phragmites australis*	22.4	\	1.0	\
121	Poaceae	*Digitaria sanguinalis*	22.1	\	0.4	\
122	Poaceae	*Elymus kamoji*	52.9	\	1.6	\
123	Poaceae	*Bromus japonicus*	26.0	\	1.2	\
124	Poaceae	*Bromus catharticus*	0.3	\	0.0	\
125	Poaceae	*Aegilops tauschii*	10.7	\	0.5	\
126	Poaceae	*Eleusine indica*	14.6	\	0.3	\
127	Poaceae	*Phleum paniculatum*	3.2	6.5	0.1	0.6
128	Poaceae	*Beckmannia syzigachne*	74.7	22.4	19.2	6.6
129	Poaceae	*Avena fatua*	21.1	28.6	1.5	5.8
130	Poaceae	*Poa annua*	29.2	8.6	0.9	0.9
131	Polygonaceae	*Polygonum aviculare*	22.7	9.5	0.8	0.5
132	Polygonaceae	*Reynoutria japonica*	0.3	\	0.0	\
133	Polygonaceae	*Persicaria lapathifolia*	36.4	\	1.4	\
134	Polygonaceae	*Persicaria orientalis*	8.8	\	0.4	\
135	Polygonaceae	*Persicaria perfoliata*	5.5	\	0.1	\
136	Polygonaceae	*Persicaria viscosa*	1.3	\	0.1	\
137	Polygonaceae	*Persicaria hydropiper*	1.0	\	0.0	\
138	Polygonaceae	*Persicaria maculosa*	0.6	\	0.0	\
139	Polygonaceae	*Persicaria lapathifolia salicifolia*	0.3	2.9	0.0	0.1
140	Polygonaceae	*Rumex dentatus*	27.3	4.3	0.5	0.2
141	Polygonaceae	*Rumex microcarpus*	5.2	\	0.1	\
142	Polygonaceae	*Rumex crispus*	1.0	\	0.0	\
143	Polygonaceae	*Rumex acetosa*	0.3	\	0.0	\

144	Portulacaceae	*Portulaca oleracea*	7.8	\	0.0	\
145	Primulaceae	*Lysimachia candida*	7.8	\	0.1	\
146	Ranunculaceae	*Ranunculus muricatus*	9.1	\	0.3	\
147	Ranunculaceae	*Ranunculus sceleratus*	1.3	\	0.0	\
148	Ranunculaceae	*Ranunculus japonicus*	0.3	\	0.0	\
149	Rosaceae	*Potentilla supina*	10.4	\	0.2	\
150	Rubiaceae	*Paederia foetida*	1.6	\	0.0	\
151	Rubiaceae	*Galium spurium*	76.0	57.1	7.4	14.7
152	Rubiaceae	*Rubia cordifolia*	0.6	\	0.0	\
153	Sapindaceae	*Koelreuteria paniculata*	1.3	\	0.0	\
154	Solanaceae	*Solanum nigrum*	1.0	\	0.0	\
155	Typhaceae	*Typha orientalis*	0.3	\	0.0	\
156	Vitaceae	*Causonis japonica*	10.7	\	0.2	\
157	Rubiaceae	*Galium tricornutum*	\	16.3	\	1.5
158	Asteraceae	*Ixeridium dentatum*	\	5.6	\	0.4

Table S2. Proportions in dominance values and species richness out of overall weeds for different plant families.

Code	Family	Proportions in dominance values (%)	Proportions in species richness (%)
1	Compositae	5.5	16.7
2	Poaceae	54.3	14.7
3	Polygonaceae	3.4	8.3
4	Fabaceae	2.9	7.1
5	Amaranthaceae	4.1	4.5
6	Brassicaceae	2.7	5.1
7	Plantaginaceae	4.0	4.5
8	Caryophyllaceae	2.2	3.8
9	Apiaceae	1.1	2.6
10	Convolvulaceae	1.9	2.6
11	Lamiaceae	0.2	2.6
12	Euphorbiaceae	0.8	2.6
13	Boraginaceae	0.2	2.6
14	Cyperaceae	0.3	2.6
15	Ranunculaceae	0.3	1.9
16	Rubiaceae	7.5	1.9
17	Oxalidaceae	0.0	1.3
18	Geraniaceae	3.1	1.3
19	Solanaceae	0.0	0.6
20	Malvaceae	0.0	0.6
21	Phytolaccaceae	0.0	0.6
22	Araceae	0.0	0.6
23	Cannabaceae	2.8	0.6
24	Equisetaceae	0.1	0.6
25	Acanthaceae	0.0	0.6
26	Typhaceae	0.0	0.6
27	Campanulaceae	0.0	0.6
28	Apocynaceae	0.8	0.6
29	Acoraceae	0.0	0.6
30	Sapindaceae	0.0	0.6
31	Moraceae	0.0	0.6
32	Portulacaceae	0.0	0.6
33	Mazaceae	1.0	0.6
34	Onagraceae	0.0	0.6
35	Rosaceae	0.2	0.6
36	Papaveraceae	0.0	0.6
37	Primulaceae	0.1	0.6
38	Lythraceae	0.0	0.6
39	Vitaceae	0.2	0.6

Table S3. Proportions in dominance values and species richness out of overall weeds for different plant genera.

Code	Genus	Proportions in dominance values (%)	Proportions in species richness (%)
1	*Persicaria*	2.0	4.5
2	*Veronica*	4.0	3.2
3	*Erigeron*	1.4	3.2
4	*Vicia*	2.4	3.2
5	*Rumex*	0.6	2.6
6	*Amaranthus*	0.1	1.9
7	*Cyperus*	0.1	1.9
8	*Ranunculus*	0.3	1.9
9	*Euphorbia*	0.3	1.9
10	*Rorippa*	0.6	1.9
11	*Ipomoea*	0.0	1.9
12	*Artemisia*	0.0	1.9
13	*Stellaria*	1.9	1.9
14	*Alopecurus*	16.2	1.9
15	*Cardamine*	0.0	1.3
16	*Lolium*	6.6	1.3
17	*Plantago*	0.0	1.3
18	*Bothriospermum*	0.1	1.3
19	*Medicago*	0.4	1.3
20	*Geranium*	3.1	1.3
21	*Bromus*	1.2	1.3
22	*Chenopodium*	2.0	1.3
23	*Setaria*	0.1	1.3
24	*Bidens*	0.2	1.3
25	*Sonchus*	0.9	1.3
26	*Youngia*	0.0	1.3
27	*Cirsium*	0.7	1.3
28	*Oxalis*	0.0	1.3
29	*Lagopsis*	0.0	0.6
30	*Cnidium*	1.1	0.6
31	*Torilis*	0.0	0.6
32	*Aeschynomene*	0.1	0.6
33	*Acalypha*	0.5	0.6
34	*Calystegia*	1.8	0.6
35	*Solidago*	0.4	0.6
36	*Oxybasis*	0.0	0.6
37	*Melilotus*	0.0	0.6
38	*Daucus*	0.0	0.6
39	*Ammannia*	0.0	0.6
40	*Reynoutria*	0.0	0.6
41	*Potentilla*	0.2	0.6
42	*Glycine*	0.1	0.6
43	*Paederia*	0.0	0.6

44	*Poa*	0.9	0.6
45	*Capsella*	1.9	0.6
46	*Trigonotis*	0.1	0.6
47	*Pinellia*	0.0	0.6
48	*Eleusine*	0.3	0.6
49	*Leersia*	1.4	0.6
50	*Salvia*	0.1	0.6
51	*Pseudosclerochloa*	0.7	0.6
52	*Broussonetia*	0.0	0.6
53	*Cerastium*	0.2	0.6
54	*Lapsanastrum*	0.1	0.6
55	*Causonis*	0.2	0.6
56	*Ludwigia*	0.0	0.6
57	*Typha*	0.0	0.6
58	*Ixeris*	0.1	0.6
59	*Lobelia*	0.0	0.6
60	*Galium*	7.4	0.6
61	*Papaver*	0.0	0.6
62	*Lepidium*	0.1	0.6
63	*Trifolium*	0.0	0.6
64	*Cynanchum*	0.8	0.6
65	*Abutilon*	0.0	0.6
66	*Eclipta*	0.0	0.6
67	*Bolboschoenus*	0.2	0.6
68	*Alternanthera*	2.0	0.6
69	*Aegilops*	0.5	0.6
70	*Symphyotrichum*	0.1	0.6
71	*Phytolacca*	0.0	0.6
72	*Polypogon*	1.8	0.6
73	*Pseudognaphalium*	0.1	0.6
74	*Phragmites*	1.0	0.6
75	*Oenanthe*	0.0	0.6
76	*Lithospermum*	0.0	0.6
77	*Cynodon*	0.3	0.6
78	*Echinochloa*	0.4	0.6
79	*Phleum*	0.1	0.6
80	*Portulaca*	0.0	0.6
81	*Mazus*	1.0	0.6
82	*Strobilanthes*	0.0	0.6
83	*Beckmannia*	19.2	0.6
84	*Digitaria*	0.4	0.6
85	*Lactuca*	0.3	0.6
86	*Polygonum*	0.8	0.6
87	*Arenaria*	0.0	0.6
88	*Equisetum*	0.1	0.6
89	*Acorus*	0.0	0.6
90	*Carduus*	0.0	0.6
91	*Helianthus*	0.0	0.6

92	*Hemisteptia*	1.3	0.6
93	*Avena*	1.5	0.6
94	*Elymus*	1.6	0.6
95	*Lamium*	0.0	0.6
96	*Descurainia*	0.2	0.6
97	*Leonurus*	0.1	0.6
98	*Sagina*	0.1	0.6
99	*Rubia*	0.0	0.6
100	*Lysimachia*	0.1	0.6
101	*Solanum*	0.0	0.6
102	*Koelreuteria*	0.0	0.6
103	*Humulus*	2.8	0.6
